# Supplementary material for: Electroacupuncture Relieves Visceral Hypersensitivity via Balancing PAR2 and PAR4 in the Descending Pain Modulatory System of Goats
Source: Brain Sci. 2023 Jun 7;13(6):922. doi: 10.3390/brainsci13060922 (PMC10296180; doi:10.3390/brainsci13060922)
Supplement: Supplementary file 1 [file brainsci-13-00922-s001.zip › supplementary materials.pdf]

Supplementary data

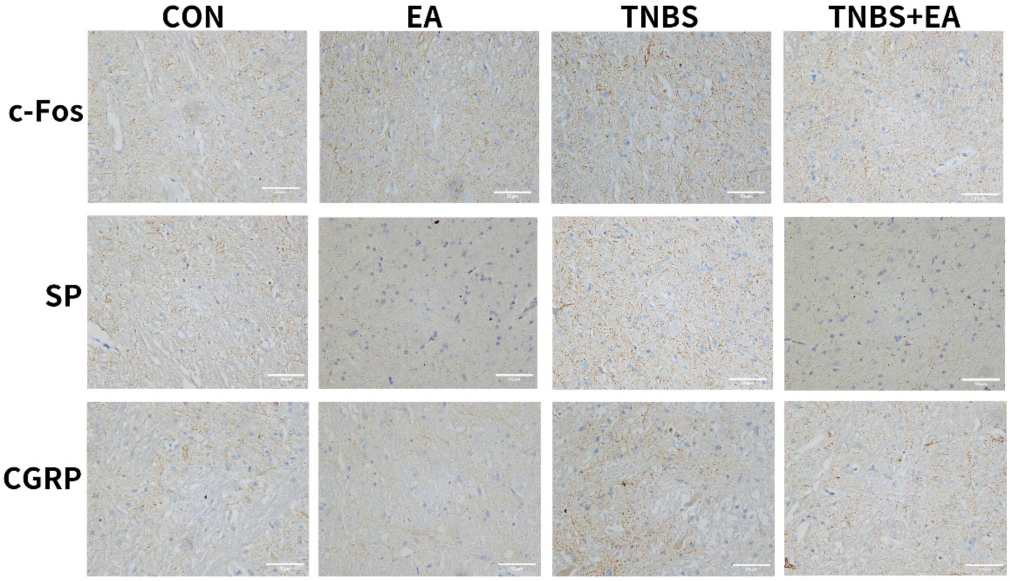

Figure S1. Immunohistochemical staining of c-Fos, SP and CGRP proteins (40×) in PAG respectively on day 25 (n = 6). Brown precipitate indicates immune-positive cells, blue stain represents nuclei. Scale bars = 50 µm.

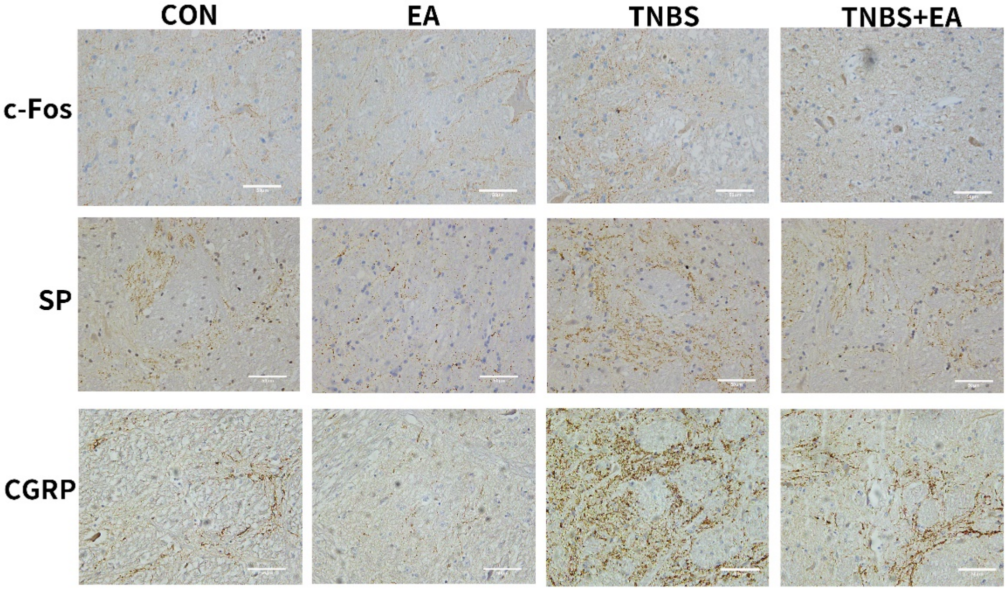

Figure S2. Immunohistochemical staining of c-Fos, SP and CGRP proteins (40×) in RVM respectively on day 25 (n = 6). Brown precipitate indicates immune-positive cells, blue stain represents nuclei. Scale bars = 50 µm.

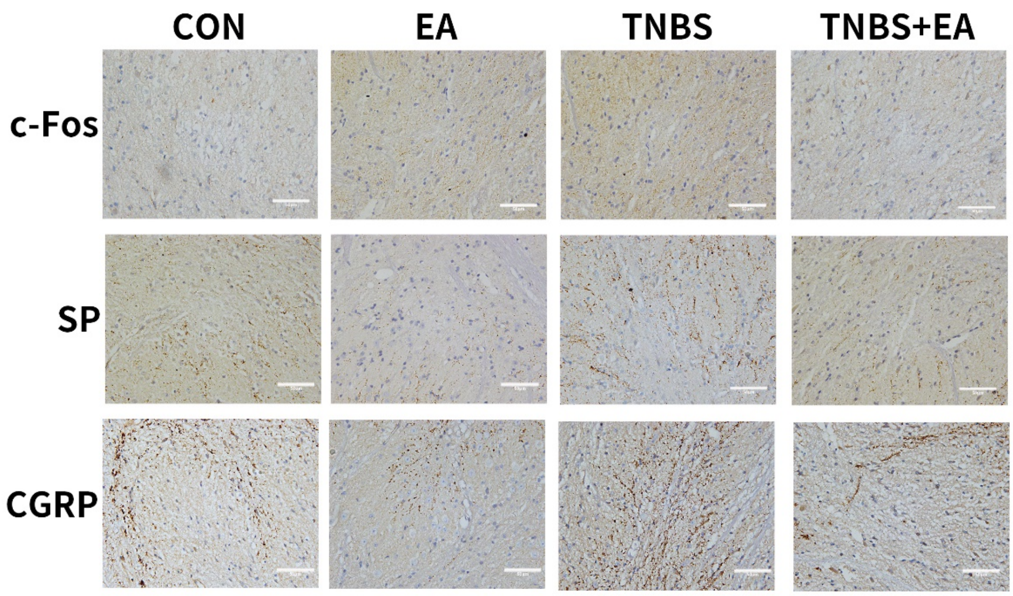

Figure S3. Immunohistochemical staining of c-Fos, SP and CGRP proteins (40×) in SCDH respectively on day 25 (n = 6). Brown precipitate indicates immune-positive cells, blue stain represents nuclei. Scale bars = 50 μm.

Table S1 Criteria for nociceptive behavioral response scores (0-4)

Scoring	Level	Description
0	Normal behavior	No signs of discomfort
1	Slightly modifiedbehavior	Rapid and shallow breathing, increased heart reat, immobility of hind limbs
2	Mild behavior	Restlessness, alertness, changed posture, tail wagging, head back to the abdomen
3	Moderate behavior	Tail wagging, head back to the abdomen, the back arching together with weight shifting, occasional grunting, and teeth grinding
4	Severe behavior	Head back to the abdomen, stomping feet or kicking the abdomen, repetitive back arching together with weight shifting, frequent grunting, and teeth grinding

Table S2 Grading criteria for ileal macroscopic and microscopic changes

Macroscopic changes		Scores	Microscopic changes		Scores
Adhesions	None	0	Crypt depth	Normal	0
	Minimum	1		<50 % reduction	1
	Involving several bowel loops	2		>50 % reduction	2
Mucosal hyperemia	Normal	0	Inflammatory cells	No infiltration	0
	Mild	1		Few scattered cells	1
	Moderate	2		Distributed but not dense	2
	Severe	3		Dense	3
Ulcers	None	0	Blood vessel congestion	Normal	0
	Ulceration <2 cm length	1		Mild	1
	Two ulcers <2 cm	2		Moderate	2
	More sites of ulceration or one >2 cm	3		Severe	3
Wall thickness	Normal	0	Ulceration	Normal	0
	50 % increase	1		Moderate	1
	100 % increase	2		Severe	2
Maximum scores		10	Maximum scores		10
